# Supplementary material for: Introducing Isotòpia: A stable isotope database for Classical Antiquity
Source: PLoS One. 2024 Jun 3;19(6):e0293717. doi: 10.1371/journal.pone.0293717 (PMC11146721; doi:10.1371/journal.pone.0293717)
Supplement: S1 Appendix — (DOCX) [file pone.0293717.s002.docx]

S2. List of scientific publications from which isotopic data is retrieved

Al-Shorman, A., & El-Khouri, L. (2011). Strontium isotope analysis of human tooth enamel from Barsinia: A late antiquity site in Northern Jordan. Archaeological and Anthropological Sciences, 3(3), 263–269. https://doi.org/10.1007/s12520-011-0065-0

Acosta, A. N., Killgrove, K., Moses, V. C., & Turner, B. L. (2019). Nourishing urban development: A palaeodietary study of Archaic Gabii, Italy (6th–5th c BCE). Journal of Archaeological Science: Reports, 27. https://doi.org/10.1016/j.jasrep.2019.101962

Aguilera, M., Zech-Matterne, V., Lepetz, S., & Balasse, M. (2018). Crop Fertility Conditions in North-Eastern Gaul During the La Tène and Roman Periods: A Combined Stable Isotope Analysis of Archaeobotanical and Archaeozoological Remains. Environmental Archaeology, 23(4), 323–337. https://doi.org/10.1080/14614103.2017.1291563

Aiken, M. (2019). Pig husbandry at kastro kallithea: An isotopic study of pig husbandry in Hellenistic Thessaly. doi:10.7939/R3-N9BR-GW95

Alagich, R., Gardeisen, A., Alonso, N., Rovira, N., & Bogaard, A. (2018). Using stable isotopes and functional weed ecology to explore social differences in early urban contexts: The case of Lattara in mediterranean France. Journal of Archaeological Science, 93, 135–149. https://doi.org/10.1016/j.jas.2018.03.006

Alagich, R., Trantalidou, K., Miller, M. C., & Smith, C. (2021). Reconstructing animal management practices at Greek Early Iron Age Zagora (Andros) using stable isotopes. Archaeological and Anthropological Sciences 13: 9. https://doi.org/10.1007/s12520-020-01249-1

Alaica, A. K., Schalburg-Clayton, J., Dalton, A., Kranioti, E., Graziani Echávarri, G., & Pickard, C. (2019). Variability along the frontier: stable carbon and nitrogen isotope ratio analysis of human remains from the Late Roman–Early Byzantine cemetery site of Joan Planells, Ibiza, Spain. Archaeological and Anthropological Sciences, 11(8), 3783–3796. https://doi.org/10.1007/s12520-018-0656-0

Al-Bashaireh, K., Al-Shorman, A., Rose, J., Timothy Jull, A. J., & Hodgins, G. (2010). Paleodiet reconstruction of human remains from the archaeo-logical site of Natfieh, northern Jordan. In j t jull radiocarbon (vol. 52). https://doi.org/10.1017/S0033822200045677

Albizuri, S., Grandal-d’Anglade, A., Maroto, J., Oliva, M., Rodríguez, A., Terrats, N., … López-Cachero, F. J. (2021). Dogs that ate plants: Changes in the canine diet during the late bronze age and the first iron age in the northeast Iberian peninsula. Journal of World Prehistory, 34(1), 75–119. doi:10.1007/s10963-021-09153-9

Albizuri, S., Valenzuela-Lamas, S., Bosch, D., Fernandez, M., & López-Cachero, F. J. (2019). Equid use and provision during the Early Iron Age in Can Roqueta (NE Iberian Peninsula). Zooarchaeological study and first strontium isotope result (87Sr/86Sr). Journal of Archaeological Science, Reports, 26(101907), 101907. doi:10.1016/j.jasrep.2019.101907

Alexander, M.M., Gutiérrez, A., Millard, A.R., Richards, M.P., Gerrard, C.M. (2019). Economic and socio-cultural consequences of changing political rule on human and faunal diets in medieval Valencia (c. fifth–fifteenth century AD) as evidenced by stable isotopes. Archaeological and Anthropological Sciences 11: 3875–3893.

Antikas, T. G. (2008). FISH EATING HORSES IN CENTRAL MACEDONIA OF THE 5 TH CENTURY BCE: WAS HERODOTUS FINALLY RIGHT? In Vet Med Zoot). T (Issue 66).

Armit, I., Fischer, C.-E., Koon, H., Nicholls, R., Olalde, I., Rohland, N., Buckberry, J., Montgomery, J., Mason, P., Črešnar, M., Büster, L., & Reich, D. (2023). Kinship practices in Early Iron Age South-east Europe: genetic and isotopic analysis of burials from the Dolge njive barrow cemetery, Dolenjska, Slovenia. In Antiquity (Vol. 97, Issue 392, pp. 403–418). Antiquity Publications. https://doi.org/10.15184/aqy.2023.2

Arnold, E. R., Greer, J. S., Ilan, D., Thareani, Y., & Hartman, G. (2021). “Come, O pilgrim”—but buy local: an isotopic investigation of animal provisioning at Iron Age II Tel Dan. Archaeological and Anthropological Sciences, 13(4). doi:10.1007/s12520-021-01291-7

Baldoni, M., Gismondi, A., Alexander, M., D’Agostino, A., Tibaldi, D., Di Marco, G., Scano, G., Canini, A., Caserta, E., Rickards, O., & Martínez-Labarga, C. (2019). A multidisciplinary approach to investigate the osteobiography of the Roman Imperial population from Muracciola Torresina (Palestrina, Rome, Italy). In Journal of Archaeological Science: Reports (Vol. 27, p. 101960). Elsevier BV. https://doi.org/10.1016/j.jasrep.2019.101960

Berger, T. E., Peters, J., & Grupe, G. (2010). Life history of a mule (c. 160 AD) from the Roman fort Biriciana/Weißenburg (Upper Bavaria) as revealed by serial stable isotope analysis of dental tissues. International Journal of Osteoarchaeology, 20(2), 158–171. https://doi.org/10.1002/oa.1013

Bonsall, L. A., & Pickard, C. (2015). Stable isotope and dental pathology evidence for diet in late Roman Winchester, England. In Journal of Archaeological Science: Reports (Vol. 2, pp. 128–140). Elsevier BV. https://doi.org/10.1016/j.jasrep.2015.01.009

Bourbou, C., Arenz, G., Dasen, V., & Lösch, S. (2019). Babes, bones, and isotopes: A stable isotope investigation on nonadults from Aventicum, Roman Switzerland (first–third century CE). International Journal of Osteoarchaeology, 29(6), 974–985. https://doi.org/10.1002/oa.2811

Brettell, R., Evans, J., Marzinzik, S., Lamb, A., & Montgomery, J. (2012). ‘Impious Easterners’: Can Oxygen and Strontium Isotopes Serve as Indicators of Provenance in Early Medieval European Cemetery Populations? In European Journal of Archaeology (Vol. 15, Issue 1, pp. 117–145). Cambridge University Press (CUP). https://doi.org/10.1179/1461957112y.0000000001

Brönnimann, D., Knipper, C., Pichler, S. L., Röder, B., Rissanen, H., Stopp, B., Rosner, M., Blank, M., Warnberg, O., Alt, K. W., Lassau, G., & Rentzel, P. (2018). The lay of land: Strontium isotope variability in the dietary catchment of the Late Iron Age proto-urban settlement of Basel-Gasfabrik, Switzerland. In Journal of Archaeological Science: Reports (Vol. 17, pp. 279–292). Elsevier BV. https://doi.org/10.1016/j.jasrep.2017.11.009

Budd, P., Millard, A., Chenery, C., Lucy, S., & Roberts, C. (2004). Investigating population movement by stable isotope analysis: a report from Britain. Antiquity, 78, 299, 127-141 https://doi.org/10.1017/S0003598X0009298X

Budd, P., Montgomery, J., Evans, J., & Chenery, C. (2007). Combined Pb, Sr- and O-isotope analysis of human dental tissue for the reconstruction of archaeological residential mobility (pp. 311–324). https://doi.org/10.1039/9781847551696-00311

Caputo, I., Lepretti, M., Scarabino, C., Esposito, C., & Proto, A. (2012). An acetic acid-based extraction method to obtain high quality Collagen from archeological bone remains. Analytical Biochemistry, 421(1), 92–96. https://doi.org/10.1016/j.ab.2011.10.024

Carver, M. (2008). Wasperton Anglo-Saxon Cemetery [Data set]. Archaeology Data Service. https://doi.org/10.5284/1000052

Castells Navarro, L., Buckberry, J., Beaumont, J. (2022). An isotope signature for diffuse idiopathic skeletal hyperostosis?. American Journal of Biological Anthropology 178: 312-327.

Cau, M. Á., Rullan, M. R., Salas, M., & van Strydonck, M. (2014). Radiocarbon Dating of the Necropolis of the Early Christian Site of Son Peretó (Mallorca, Balearic Islands). Radiocarbon, 56(2), 399–410. https://doi.org/10.2458/56.17458

Chenery, C., Eckardt, H., & Müldner, G. (2011). Cosmopolitan Catterick? Isotopic evidence for population mobility on Rome’s Northern frontier. Journal of Archaeological Science, 38(7), 1525–1536. https://doi.org/10.1016/j.jas.2011.02.018

Chenery, C., Müldner, G., Evans, J., Eckardt, H., & Lewis, M. (2010). Strontium and stable isotope evidence for diet and mobility in Roman Gloucester, UK. Journal of Archaeological Science, 37(1), 150–163. https://doi.org/10.1016/j.jas.2009.09.025

Cheung, C., Schroeder, H., & Hedges, R. E. M. (2012). Diet, social differentiation and cultural change in Roman Britain: New isotopic evidence from Gloucestershire. Archaeological and Anthropological Sciences, 4(1), 61–73. https://doi.org/10.1007/s12520-011-0083-y

Clauzel, T., Richardin, P., Ricard, J., Le Béchennec, Y., Amiot, R., Fourel, F., Phouybanhdyt, B., Joseph, D., Vinçon-Laugier, A., Flandrois, J.-P., & Lécuyer, C. (2022). Climate conditions and dietary practices during the Second Iron Age studied through the multi-isotope analysis of bones and teeth from individuals of Thézy-Glimont, Picardie, France. In Archaeological and Anthropological Sciences (Vol. 14, Issue 4). Springer Science and Business Media LLC. https://doi.org/10.1007/s12520-022-01534-1

Clauzel, T., Richardin, P., Ricard, J., le Béchennec, Y., Amiot, R., Fourel, F., Phouybanhdyt, B., Vinçon-Laugier, A., Flandrois, J. P., & Lécuyer, C. (2020). The Gauls experienced the Roman Warm Period: Oxygen isotope study of the Gallic site of Thézy-Glimont, Picardie, France. Journal of Archaeological Science: Reports, 34. https://doi.org/10.1016/j.jasrep.2020.102595

Cocozza, C., Fernandes, R., Ughi, A., Groß, M., & Alexander, M. M. (2021). Investigating infant feeding strategies at Roman Bainesse through Bayesian modelling of incremental dentine isotopic data. International Journal of Osteoarchaeology, 31(3), 429–439. https://doi.org/10.1002/oa.2962

Craig, O. E., Biazzo, M., O’Connell, T. C., Garnsey, P., Martinez-Labarga, C., Lelli, R., Salvadei, L., Tartaglia, G., Nava, A., Renò, L., Fiammenghi, A., Rickards, O., & Bondioli, L. (2009). Stable isotopic evidence for diet at the imperial roman coastal site of Velia (1st and 2nd centuries AD) in Southern Italy. American Journal of Physical Anthropology, 139(4), 572–583. https://doi.org/10.1002/ajpa.21021

Craig, O. E., Bondioli, L., Fattore, L., Higham, T., & Hedges, R. (2013). Evaluating marine diets through radiocarbon dating and stable isotope analysis of victims of the AD 79 eruption of vesuvius. American Journal of Physical Anthropology, 152(3), 345–352. https://doi.org/10.1002/ajpa.22352

Crowder, K. D., Montgomery, J., Filipek, K. L., & Evans, J. A. (2020). Romans, barbarians and foederati: New biomolecular data and a possible region of origin for “Headless Romans” and other burials from Britain. Journal of Archaeological Science: Reports, 30. https://doi.org/10.1016/j.jasrep.2019.102180

Crowder, K. D., Montgomery, J., Gröcke, D. R., & Filipek, K. L. (2019). Childhood “stress” and stable isotope life histories in Transylvania. International Journal of Osteoarchaeology, 29(4), 644–653. https://doi.org/10.1002/oa.2760

Crowe, F., Sperduti, A., O’Connell, T. C., Craig, O. E., Kirsanow, K., Germoni, P., Macchiarelli, R., Garnsey, P., & Bondioli, L. (2010). Water-related occupations and diet in two Roman coastal communities (Italy, first to third century AD): Correlation between stable carbon and nitrogen isotope values and auricular exostosis prevalence. American Journal of Physical Anthropology, 142(3), 355–366. https://doi.org/10.1002/ajpa.21229

Czermak, A., Schermelleh, L., & Lee-Thorp, J. (2018). Imaging-assisted time-resolved dentine sampling to track weaning histories. In International Journal of Osteoarchaeology (Vol. 28, Issue 5, pp. 535–541). Wiley. https://doi.org/10.1002/oa.2697

Czermak, A., Schermelleh, L., & Lee-Thorp, J. (2019). Fluorescence screening of collagen preservation in tooth dentine. In Palaeogeography, Palaeoclimatology, Palaeoecology (Vol. 532, p. 109249). Elsevier BV. https://doi.org/10.1016/j.palaeo.2019.109249

de Angelis, F., Varano, S., Battistini, A., di Giannantonio, S., Ricci, P., Lubritto, C., Facchin, G., Brancazi, L., Santangeli-Valenzani, R., Catalano, P., Gazzaniga, V., Rickards, O., & Martínez-Labarga, C. (2020). Food at the heart of the Empire: dietary reconstruction for Imperial Rome inhabitants. Archaeological and Anthropological Sciences, 12(10). https://doi.org/10.1007/s12520-020-01194-z

de Angelis, F., Veltre, V., Varano, S., Romboni, M., Renzi, S., Zingale, S., Ricci, P., Caldarini, C., Giannantonio, S. di, Lubritto, C., Catalano, P., Rickards, O., & Martínez-Labarga, C. (2020). Dietary and Weaning Habits of the Roman Community of Quarto Cappello del Prete (Rome, 1st-3rd Century CE). Environmental Archaeology. https://doi.org/10.1080/14614103.2020.1829297

Dotsika, E., & Michael, D. E. (2018). Using stable isotope technique in order to assess the dietary habits of a Roman population in Greece. Journal of Archaeological Science: Reports, 22, 470–481. https://doi.org/10.1016/j.jasrep.2018.04.015

Dotsika, E., Tassi, M., Karalis, P., Chrysostomou, A., Michael, D. E., Poutouki, A. E., Theodorakopoulou, K., & Diamantopoulos, G. (2022). Stable Isotope and Radiocarbon Analysis for Diet, Climate and Mobility Reconstruction in Agras (Early Iron Age) and Edessa (Roman Age), Northern Greece. Applied Sciences (Switzerland), 12(1). https://doi.org/10.3390/app12010498

Dupras, T. L., Schwarcz, H. P., & Fairgrieve, S. I. (2001). Infant Feeding and Weaning Practices in Roman Egypt. In J Phys Anthropol (Vol. 115). White and Schwarcz. https://doi.org/10.1002/ajpa.1075

Dupras, T.L. and Schwarcz, H.P., 2001. Strangers in a strange land: stable isotope evidence for human migration in the Dakhleh Oasis, Egypt. Journal of Archaeological Science, 28(11), pp.1199-1208. https://doi.org/10.1006/jasc.2001.0640

Dupras, T.L. and Tocheri, M.W., 2007. Reconstructing infant weaning histories at Roman period Kellis, Egypt using stable isotope analysis of dentition. American Journal of Physical Anthropology: The Official Publication of the American Association of Physical Anthropologists, 134(1), pp.63-74. https://doi.org/10.1002/ajpa.20639

Eckardt, H., Chenery, C., Booth, P., Evans, J. A., Lamb, A., & Müldner, G. (2009). Oxygen and strontium isotope evidence for mobility in Roman Winchester. Journal of Archaeological Science, 36(12), 2816–2825. https://doi.org/10.1016/j.jas.2009.09.010

Eckardt, H., Müldner, G., & Speed, G. (2015). The Late Roman Field Army in Northern Britain? Mobility, Material Culture and Multi-Isotope Analysis at Scorton (N Yorks.). Britannia, 46, 191–223. https://doi.org/10.1017/S0068113X1500015X

Emery, M. v., Duggan, A. T., Murchie, T. J., Stark, R. J., Klunk, J., Hider, J., Eaton, K., Karpinski, E., Schwarcz, H. P., Poinar, H. N., & Prowse, T. L. (2018). Ancient Roman mitochondrial genomes and isotopes reveal relationships and geographic origins at the local and pan-Mediterranean scales. Journal of Archaeological Science: Reports, 20, 200–209. https://doi.org/10.1016/j.jasrep.2018.04.036

Emery, M. v., Stark, R. J., Murchie, T. J., Elford, S., Schwarcz, H. P., & Prowse, T. L. (2018). Mapping the origins of Imperial Roman workers (1st–4th century CE) at Vagnari, Southern Italy, using 87Sr/86Sr and δ18O variability. American Journal of Physical Anthropology, 166(4), 837–850. https://doi.org/10.1002/ajpa.23473

Ervynck, A., Boudin, M., van den Brande, T., & van Strydonck, M. (2014). Dating Human Remains from the Historical Period in Belgium: Diet Changes and the Impact of Marine and Freshwater Reservoir Effects. Radiocarbon, 56(2), 779–788. https://doi.org/10.1017/S003382220004981X

Eshel, T., Yahalom-Mack, N., Tirosh, O., Maeir, A. M., Harlavan, Y., Gilboa, A., & Erel, Y. (2020). Pollution and human mobility in the southern Levant during the Iron Age using chemical and isotopic analysis of human tooth enamel. In Journal of Archaeological Science (Vol. 124, p. 105262). Elsevier BV. https://doi.org/10.1016/j.jas.2020.105262

Esposito, C., Gigante, M., Lugli, F., Miranda, P., Cavazzuti, C., Sperduti, A., ... & Müller, W. (2023). Intense community dynamics in the pre-Roman frontier site of Fermo (ninth–fifth century BCE, Marche, central Italy) inferred from isotopic data. Scientific Reports, 13(1), 3632.

Evans, J. A., Tatham, S., Chenery, S. R., & Chenery, C. A. (2007). Anglo-Saxon animal husbandry techniques revealed though isotope and chemical variations in cattle teeth. In Applied Geochemistry (Vol. 22, Issue 9, pp. 1994–2005). Elsevier BV. https://doi.org/10.1016/j.apgeochem.2007.03.059

Fernandes, D., Sirak, K., Cheronet, O., Howcroft, R., Čavka, M., Los, D., Burmaz, J., Pinhasi, R., & Novak, M. (2019). Cranial deformation and genetic diversity in three adolescent male individuals from the Great Migration Period from Osijek, eastern Croatia. In P. F. Biehl (Ed.), PLOS ONE (Vol. 14, Issue 8, p. e0216366). Public Library of Science (PLoS). https://doi.org/10.1371/journal.pone.0216366

Fernández-Crespo, T., Ordoño, J., Bogaard, A., Llanos, A., & Schulting, R. (2019). A snapshot of subsistence in Iron Age Iberia: The case of La Hoya village. In Journal of Archaeological Science: Reports (Vol. 28, p. 102037). Elsevier BV. https://doi.org/10.1016/j.jasrep.2019.102037

Fernandez-Martinez, P., Maurer, A. F., Jiménez-Morillo, N. T., Botella, M., Lopez, B., & Barrocas Dias, C. (2020). Bone stable isotope data of the Late Roman population (4th–7th centuries CE) from Mondragones (Granada): A dietary reconstruction in a Roman villa context of south-eastern Spain. Journal of Archaeological Science: Reports, 33. https://doi.org/10.1016/j.jasrep.2020.102566

Fiorin, E., Moore, J., Montgomery, J., Lippi, M. M., Nowell, G., & Forlin, P. (2021). Combining dental calculus with isotope analysis in the Alps: New evidence from the Roman and medieval cemeteries of Lamon, Italy. Quaternary International. https://doi.org/10.1016/j.quaint.2021.11.022

Frémondeau, D., de Cupere, B., Evin, A., & van Neer, W. (2017). Diversity in pig husbandry from the Classical-Hellenistic to the Byzantine periods: An integrated dental analysis of Düzen Tepe and Sagalassos assemblages (Turkey). Journal of Archaeological Science: Reports, 11, 38–52. https://doi.org/10.1016/J.JASREP.2016.11.030

Frémondeau, D., Nuviala, P., & Duval, C. (2017). Pigs and cattle in Gaul: the role of Gallic societies in the evolution of husbandry practices. European Journal of Archaeology, 20(3), 494-509.

Fuller, B. T., de Cupere, B., Marinova, E., van Neer, W., Waelkens, M., & Richards, M. P. (2012). Isotopic reconstruction of human diet and animal husbandry practices during the Classical-Hellenistic, imperial, and Byzantine periods at Sagalassos, Turkey. American Journal of Physical Anthropology, 149(2), 157–171. https://doi.org/10.1002/ajpa.22100

Fuller, B. T., Márquez-Grant, N., & Richards, M. P. (2010). Investigation of diachronic dietary patterns on the islands of Ibiza and formentera, Spain: Evidence from carbon and nitrogen stable isotope ratio analysis. In American Journal of Physical Anthropology (Vol. 143, Issue 4, pp. 512–522). https://doi.org/10.1002/ajpa.21334

Fuller, B. T., Molleson, T. I., Harris, D. A., Gilmour, L. T., & Hedges, R. E. M. (2006). Isotopic evidence for breastfeeding and possible adult dietary differences from Late/Sub-Roman Britain. American Journal of Physical Anthropology, 129(1), 45–54. https://doi.org/10.1002/ajpa.20244

Fuller, B. T., Van Neer, W., Linseele, V., De Cupere, B., Chahoud, J., & Richards, M. P. (2020). Fish δ13C and δ15N results from two Bronze/Iron Age sites (Tell Tweini & Sidon) along the Levantine coast. Journal of Archaeological Science, Reports, 29(102066), 102066. doi:10.1016/j.jasrep.2019.102066

Gan, Y. M., Towers, J., Bradley, R. A., Pearson, E., Nowell, G., Peterkin, J., & Montgomery, J. (2018). Multi-isotope evidence for cattle droving at Roman Worcester. Journal of Archaeological Science: Reports, 20, 6–17. https://doi.org/10.1016/j.jasrep.2018.03.028

García-Collado, M. I., Ricci, P., Catalán Ramos, R., Altieri, S., Lubritto, C., & Quirós Castillo, J. A. (2018). Palaeodietary reconstruction as an alternative approach to poorly preserved early medieval human bone assemblages: the case of Boadilla (Toledo, Spain). In Archaeological and Anthropological Sciences (Vol. 11, Issue 8, pp. 3765–3782). Springer Science and Business Media LLC. https://doi.org/10.1007/s12520-018-0672-0

González-Rabanal, B., Marín-Arroyo, A. B., Jones, J. R., Pérez, L. A., Vega-Maeso, C., & González-Morales, M. R. (2020). Diet, mobility and death of Late Neolithic and Chalcolithic groups of the Cantabrian Region (northern Spain). A multidisciplinary approach towards studying the Los Avellanos I and II burial caves. Journal of Archaeological Science: Reports, 34, 102644.

Goude, G., Balasescu, A., Réveillas, H., Thomas, Y., & Lefranc, P. (2014). Diet Variability and Stable Isotope Analyses: Looking for Variables Within the Late Neolithic and Iron Age Human Groups from Gougenheim Site and Surrounding Areas (Alsace, France). In International Journal of Osteoarchaeology (Vol. 25, Issue 6, pp. 988–996). Wiley. https://doi.org/10.1002/oa.2399

Goude, G., Dori, I., Sparacello, V. S., Starnini, E., & Varalli, A. (2020). Multi-proxy stable isotope analyses of dentine microsections reveal diachronic changes in life history adaptations, mobility, and tuberculosis-induced wasting in prehistoric Liguria (Finale Ligure, Italy, northwestern Mediterranean). In International Journal of Paleopathology (Vol. 28, pp. 99–111). Elsevier BV. https://doi.org/10.1016/j.ijpp.2019.12.007

Grandal-d’Anglade, A., Albizuri, S., & López-Cachero, F. J. (2021). Equine diet during protohistoric times in the Northeast of the Iberian Peninsula: Stable isotope data (C, N) from bone collagen. Data in Brief, 38(107374), 107374. doi:10.1016/j.dib.2021.107374

Gregoricka, L. A., & Sheridan, S. G. (2013). Ascetic or affluent? Byzantine diet at the monastic community of St. Stephen’s, Jerusalem from stable carbon and nitrogen isotopes. Journal of Anthropological Archaeology, 32(1), 63–73. https://doi.org/10.1016/J.JAA.2012.10.002

Groot, M., Albarella, U., Eger, J., & Evans, J. (2021). Cattle management in an Iron Age/Roman settlement in the Netherlands: Archaeozoological and stable isotope analysis. PLoS ONE, 16(10 October). https://doi.org/10.1371/journal.pone.0258234

Groot, M., Evans, J., & Albarella, U. (2020). Mobility of cattle in the Iron Age and Roman Netherlands. In Journal of Archaeological Science: Reports (Vol. 32, p. 102416). Elsevier BV. https://doi.org/10.1016/j.jasrep.2020.102416

Gulyás, S., Balogh, C., Marcsik, A., & Sümegi, P. (2018). Simple Calibration versus Bayesian Modeling of Archeostatigraphically Controlled 14C Ages in an Early Avar Age Cemetery from SE Hungary: Results, Advantages, Pitfalls. In Radiocarbon (Vol. 60, Issue 5, pp. 1335–1346). Cambridge University Press (CUP). https://doi.org/10.1017/rdc.2018.116

Guy, S.-V., Thomas, T., Irit, Z., Andreas, P., Dorit, S., Omri, L., … Guy, B.-O. (2018). Tooth oxygen isotopes reveal Late Bronze Age origin of Mediterranean fish aquaculture and trade. Scientific Reports, 8(1), 14086. doi:10.1038/s41598-018-32468-1

Habinger, S. G., de Cupere, B., Dövener, F., Pucher, E., & Bocherens, H. (2020). Mobility and origin of camels in the Roman Empire through serial stable carbon and oxygen isotope variations in tooth enamel. Quaternary International, 557, 80–91. https://doi.org/10.1016/j.quaint.2020.05.029

Hakenbeck, S. E., Evans, J., Chapman, H., & Fóthi, E. (2017). Practising pastoralism in an agricultural environment: An isotopic analysis of the impact of the Hunnic incursions on Pannonian populations. PLoS ONE, 12(3). https://doi.org/10.1371/journal.pone.0173079

Hakenbeck, S., McManus, E., Geisler, H., Grupe, G., & O’Connell, T. (2010). Diet and mobility in early medieval bavaria: A study of carbon and nitrogen stable isotopes. American Journal of Physical Anthropology, 143(2), 235–249. https://doi.org/10.1002/ajpa.21309

Heaton, T. H. E., Jones, G., Halstead, P., & Tsipropoulos, T. (2009). Variations in the 13C/12C ratios of modern wheat grain, and implications for interpreting data from Bronze Age Assiros Toumba, Greece. Journal of Archaeological Science, 36(10), 2224–2233. https://doi.org/10.1016/j.jas.2009.06.007

Hemer, K. A., Evans, J. A., Chenery, C. A., & Lamb, A. L. (2013). Evidence of early medieval trade and migration between Wales and the Mediterranean Sea region. In Journal of Archaeological Science (Vol. 40, Issue 5, pp. 2352–2359). Elsevier BV. https://doi.org/10.1016/j.jas.2013.01.014

Hemer, K. A., Lamb, A. L., Chenery, C. A., & Evans, J. A. (2016). A multi-isotope investigation of diet and subsistence amongst island and mainland populations from early medieval western Britain. In American Journal of Physical Anthropology (Vol. 162, Issue 3, pp. 423–440). Wiley. https://doi.org/10.1002/ajpa.23127

Holland, G., Tanner, S. D., Budd, P., Chenery, C., Montgomery, J., Evans, J., & Powlesland, D. (2003). Anglo-Saxon residential mobility at West Heslerton, North Yorkshire, UK from Combined O- and SR-isotope analysis. In Plasma Source Mass Spectrometry (pp. 195–208). The Royal Society of Chemistry. https://doi.org/10.1039/9781847551689-00195

Hughes, S. S., Millard, A. R., Chenery, C. A., Nowell, G., & Pearson, D. G. (2018). Isotopic analysis of burials from the early Anglo-Saxon cemetery at Eastbourne, Sussex, U.K. In Journal of Archaeological Science: Reports (Vol. 19, pp. 513–525). Elsevier BV. https://doi.org/10.1016/j.jasrep.2018.03.004

Hughes, S. S., Millard, A. R., Lucy, S. J., Chenery, C. A., Evans, J. A., Nowell, G., & Pearson, D. G. (2014). Anglo-Saxon origins investigated by isotopic analysis of burials from Berinsfield, Oxfordshire, UK. In Journal of Archaeological Science (Vol. 42, pp. 81–92). Elsevier BV. https://doi.org/10.1016/j.jas.2013.10.025

Inskip, S. A., Taylor, G. M., Zakrzewski, S. R., Mays, S. A., Pike, A. W. G., Llewellyn, G., Williams, C. M., Lee, O. Y.-C., Wu, H. H. T., Minnikin, D. E., Besra, G. S., & Stewart, G. R. (2015). Osteological, Biomolecular and Geochemical Examination of an Early Anglo-Saxon Case of Lepromatous Leprosy. In M. Spigelman (Ed.), PLOS ONE (Vol. 10, Issue 5, p. e0124282). Public Library of Science (PLoS). https://doi.org/10.1371/journal.pone.0124282

Jay, M. (2008). Iron Age Diet at Glastonbury Lake Village: The isotopic evidence for negligible aquatic resource consumption. Oxford Journal of Archaeology, 27(2), 201–216. https://doi.org/10.1111/J.1468-0092.2008.00304.X

Jay, M., & Richards, M. P. (2006). Diet in the Iron Age cemetery population at Wetwang Slack, East Yorkshire, UK: Carbon and nitrogen stable isotope evidence. Journal of Archaeological Science, 33(5), 653–662. https://doi.org/10.1016/j.jas.2005.09.020

Jay, M., Fuller, B. T., Richards, M. P., Knüsel, C. J., & King, S. S. (2008). Iron age breastfeeding practices in Britain: Isotopic evidence from wetwang slack, east yorkshire. American Journal of Physical Anthropology, 136(3), 327–337. https://doi.org/10.1002/ajpa.20815

Jay, M., Montgomery, J., Nehlich, O., Towers, J., & Evans, J. (2013). British Iron Age chariot burials of the Arras culture: a multi-isotope approach to investigating mobility levels and subsistence practices. World Archaeology, 45(3), 473–491. https://doi.org/10.1080/00438243.2013.820647

Jernejčič, B. Š., & Price, T. D. (2020). Isotopic investigations of human cremations from the late bronze age/early iron age cemetery of ljubljana–dvorišče sazu, slovenia. Journal of Archaeological Science: Reports, 34, 102594.

Jordana, X., Malgosa, A., Casté, B., & Tornero, C. (2019). Lost in transition: the dietary shifts from Late Antiquity to the Early Middle Ages in the North Eastern Iberian Peninsula. In Archaeological and Anthropological Sciences (Vol. 11, Issue 8, pp. 3751–3763). Springer Science and Business Media LLC. https://doi.org/10.1007/s12520-019-00777-9

Kaal, J., López-Costas, O., & Martínez Cortizas, A. (2016). Diagenetic effects on pyrolysis fingerprints of extracted collagen in archaeological human bones from NW Spain, as determined by pyrolysis-GC-MS. In Journal of Archaeological Science (Vol. 65, pp. 1–10). Elsevier BV. https://doi.org/10.1016/j.jas.2015.11.001

Keenleyside, A., Schwarcz, H. P., & Panayotova, K. (2011). Oxygen isotopic evidence of residence and migration in a Greek colonial population on the Black Sea. Journal of Archaeological Science, 38(10), 2658–2666. https://doi.org/10.1016/j.jas.2011.06.001

Keenleyside, A., Schwarcz, H., & Panayotova, K. (2006). Stable isotopic evidence of diet in a Greek colonial population from the Black Sea. Journal of Archaeological Science, 33(9), 1205–1215. https://doi.org/10.1016/j.jas.2005.12.008

Kendall, E. J., Millard, A., Beaumont, J., Gowland, R., Gorton, M., & Gledhill, A. (2019). What Doesn’t Kill You: Early Life Health and Nutrition in Early Anglo-Saxon East Anglia. In The Mother-Infant Nexus in Anthropology (pp. 103–123). Springer International Publishing. https://doi.org/10.1007/978-3-030-27393-4_6

Killgrove, K., & Montgomery, J. (2016). All roads lead to Rome: Exploring human migration to the eternal city through biochemistry of skeletons from two imperial-era cemeteries (1st-3rd c AD). PLoS ONE, 11(2). https://doi.org/10.1371/journal.pone.0147585

Killgrove, K., & Tykot, R. H. (2013). Food for Rome: A stable isotope investigation of diet in the Imperial period (1st-3rd centuries AD). Journal of Anthropological Archaeology, 32(1), 28–38. https://doi.org/10.1016/j.jaa.2012.08.002

Killgrove, K., & Tykot, R. H. (2018). Diet and collapse: A stable isotope study of Imperial-era Gabii (1st–3rd centuries AD). Journal of Archaeological Science: Reports, 19, 1041–1049. https://doi.org/10.1016/j.jasrep.2017.05.054

Knipper, C., Held, P., Fecher, M., Nicklisch, N., Meyer, C., Schreiber, H., Zich, B., Metzner-Nebelsick, C., Hubensack, V., Hansen, L., Nieveler, E., Alt, K.W. (2015). Superior in Life-Superior in Death. Dietary Distinction of Central European Prehistoric and Medieval Elites. Current Anthropology 56: 579-589

Knipper, C., Koncz, I., Ódor, J. G., Mende, B. G., Rácz, Z., Kraus, S., van Gyseghem, R., Friedrich, R., & Vida, T. (2020). Coalescing traditions—Coalescing people: Community formation in Pannonia after the decline of the Roman Empire. In P. F. Biehl (Ed.), PLOS ONE (Vol. 15, Issue 4, p. e0231760). Public Library of Science (PLoS). https://doi.org/10.1371/journal.pone.0231760

Knipper, C., Pichler, S. L., Brönnimann, D., Rissanen, H., Rosner, M., Spichtig, N., ... & Alt, K. W. (2018). A knot in a network: Residential mobility at the Late Iron Age proto-urban centre of Basel-Gasfabrik (Switzerland) revealed by isotope analyses. Journal of Archaeological Science: Reports, 17, 735-753.

Knipper, C., Pichler, S. L., Rissanen, H., Stopp, B., Kühn, M., Spichtig, N., Röder, B., Schibler, J., Lassau, G., & Alt, K. W. (2017). What is on the menu in a Celtic town? Iron Age diet reconstructed at Basel-Gasfabrik, Switzerland. Archaeological and Anthropological Sciences, 9(7), 1307–1326. https://doi.org/10.1007/s12520-016-0362-8

Kontopoulos, I., & Sampson, A. (2015). Prehistoric diet on the island of Euboea, Greece: an isotopic investigation. Mediterranean Archaeology and Archaeometry, 15(3), 97–111. https://doi.org/10.5281/ZENODO.18361

Kwok, C. S. (2015). Moving Beyond Childhood: Reconstructing Dietary Life Histories of Bronze Age and Byzantine Greeks Using Stable Isotope Analysis of Dental and Skeletal Remains. https://doi.org/10.11575/PRISM/27019

Kwok, C. S., Garvie-Lok, S., & Katzenberg, M. A. (2018). Exploring variation in infant feeding practices in Byzantine Greece using stable isotope analysis of dentin serial sections. In International Journal of Osteoarchaeology (Vol. 28, Issue 5, pp. 563–578). Wiley. https://doi.org/10.1002/oa.2690

Laffranchi, Z., Cavalieri Manasse, G., Salzani, L., & Milella, M. (2019). Patterns of funerary variability, diet, and developmental stress in a Celtic population from NE Italy (3rd-1st c BC). In L. Bondioli (Ed.), PLOS ONE (Vol. 14, Issue 4, p. e0214372). Public Library of Science (PLoS). https://doi.org/10.1371/journal.pone.0214372

Laffranchi, Z., Granados‐Torres, A., Lösch, S., Zink, A., Dori, I., Delgado‐Huertas, A., & Milella, M. (2022). “Celts” up and down the Alps. Insights on mobility patterns in the pre‐Roman /Celtic population from Verona ( NE Italy, 3rd–1st c. BCE ): A multi‐isotopic approach . American Journal of Biological Anthropology, 178(3), 513–529. https://doi.org/10.1002/ajpa.24523

Laffranchi, Z., Huertas, A. D., Jiménez Brobeil, S. A., Torres, A. G., & Riquelme Cantal, J. A. (2016). Stable C &n isotopes in 2100 Year-B.P. human bone collagen indicate rare dietary dominance of C4 plants in NE-Italy. Scientific Reports, 6. https://doi.org/10.1038/srep38817

Laffranchi, Z., Jiménez-Brobeil, S. A., Delgado-Huertas, A., Granados-Torres, A., & Miranda, M. T. (2018). Infant feeding practices in a pre-Roman/Celtic population from Verona (Italy). Journal of Archaeological Science: Reports, 17, 30–38. https://doi.org/10.1016/j.jasrep.2017.10.040

Leach, S., Eckardt, H., Chenery, C., Müldner, G., & Lewis, & M. (2010). A Lady of York: migration, ethnicity and identity in Roman Britain. https://doi.org/10.1017/S0003598X00099816

Leach, S., Lewis, M., Chenery, C., Müldner, G., & Eckardt, H. (2009). Migration and diversity in Roman Britain: A multidisciplinary approach to the identification of immigrants in Roman York, England. American Journal of Physical Anthropology, 140(3), 546–561. https://doi.org/10.1002/ajpa.21104

Leggett, S. (2021). Migration and cultural integration in the early medieval cemetery of Finglesham, Kent, through stable isotopes. In Archaeological and Anthropological Sciences (Vol. 13, Issue 10). Springer Science and Business Media LLC. https://doi.org/10.1007/s12520-021-01429-7

Leslie, B. G. (2012). Residential Mobility in the Rural Greek Past: A Strontium Isotope Investigation. [Master Thesis. University of Alberta]. https://doi.org/10.7939/R30H7P

Lightfoot, E., O, T. C., Stevens, R. E., Hamilton, J., Hey, G., & Hedges, R. E. (2009). An investigation into diet at the site of Yarnton, Oxfordshire, using stable carbon and nitrogen isotopes. Oxford Journal of Archaeology, 28,3: 301-322. https://doi.org/10.1111/j.1468-0092.2009.00330.x

Lightfoot, E., Ŝlaus, M., & O’Connell, T. C. (2012). Changing cultures, changing cuisines: Cultural transitions and dietary change in iron age, roman, and early medieval Croatia. American Journal of Physical Anthropology, 148(4), 543–556. https://doi.org/10.1002/ajpa.22070

Lightfoot, E., Šlaus, M., & O’Connell, T. C. (2014). Water consumption in Iron Age, Roman, and Early Medieval Croatia. American Journal of Physical Anthropology, 154(4), 535–543. https://doi.org/10.1002/ajpa.22544

Lodwick, L., Campbell, G., Crosby, V., & Müldner, G. (2021). Isotopic Evidence for Changes in Cereal Production Strategies in Iron Age and Roman Britain. Environmental Archaeology, 26(1), 13–28. https://doi.org/10.1080/14614103.2020.1718852

López-Costas, O., & Müldner, G. (2016). Fringes of the empire: Diet and cultural change at the Roman to post-Roman transition in NW Iberia. American Journal of Physical Anthropology, 161(1), 141–154. https://doi.org/10.1002/ajpa.23016

Lösch, S., Moghaddam, N., Grossschmidt, K., Risser, D. U., & Kanz, F. (2014). Stable isotope and trace element studies on gladiators and contemporary romans from Ephesus (Turkey, 2nd and 3rd Ct. AD) - Implications for differences in diet. PLoS ONE, 9(10). https://doi.org/10.1371/journal.pone.0110489

Lubritto, C., García-Collado, M. I., Ricci, P., Altieri, S., Sirignano, C., & Quirós Castillo, J. A. (2017). New Dietary Evidence on Medieval Rural Communities of the Basque Country (Spain) and Its Surroundings from Carbon and Nitrogen Stable Isotope Analyses: Social Insights, Diachronic Changes and Geographic Comparison. In International Journal of Osteoarchaeology (Vol. 27, Issue 6, pp. 984–1002). Wiley. https://doi.org/10.1002/oa.2610

Ma, Y., Bockmann, R., Stevens, S. T., Roudesli‐Chebbi, S., Amaro, A., Brozou, A., Fuller, B. T., & Mannino, M. A. (2021). Isotopic reconstruction of diet at the Vandalic period (ca. 5th–6th centuries AD) Theodosian Wall cemetery at Carthage, Tunisia. In International Journal of Osteoarchaeology (Vol. 31, Issue 3, pp. 393–405). Wiley. https://doi.org/10.1002/oa.2958

Madgwick, R., Lewis, J., Grimes, V., & Guest, P. (2019). On the hoof: exploring the supply of animals to the Roman legionary fortress at Caerleon using strontium (87Sr/86Sr) isotope analysis. Archaeological and Anthropological Sciences, 11(1), 223–235. https://doi.org/10.1007/s12520-017-0539-9

Madgwick, R., Sykes, N., Miller, H., Symmons, R., Morris, J., & Lamb, A. (2013). Fallow deer (Dama dama dama) management in Roman South-East Britain. Archaeological and Anthropological Sciences, 5(2), 111–122. https://doi.org/10.1007/s12520-013-0120-0

Martyn, R. E. V., Garnsey, P., Fattore, L., Petrone, P., Sperduti, A., Bondioli, L., & Craig, O. E. (2018). Capturing Roman dietary variability in the catastrophic death assemblage at Herculaneum. Journal of Archaeological Science: Reports, 19, 1023–1029. https://doi.org/10.1016/j.jasrep.2017.08.008

Mas Florit, C., Cau Ontiveros, M. Á., Van Strydonck, M., Boudin, M., Cardona, F., & Munar, S. (2020). RADIOCARBON DATING OF A LATE ANTIQUE NECROPOLIS FROM FELANITX (MALLORCA, BALEARIC ISLANDS). In Radiocarbon (Vol. 63, Issue 2, pp. 727–739). Cambridge University Press (CUP). https://doi.org/10.1017/rdc.2020.122

Mays, S., & Beavan, N. (2012). An investigation of diet in early Anglo-Saxon England using carbon and nitrogen stable isotope analysis of human bone collagen. In Journal of Archaeological Science (Vol. 39, Issue 4, pp. 867–874). Elsevier BV. https://doi.org/10.1016/j.jas.2011.10.013

McConnan Borstad, C., Garvie-Lok, S., & Katsonopoulou, D. (2018). Diet at ancient Helike, Achaea, Greece based on stable isotope analysis: From the Hellenistic to the Roman and Byzantine periods. Journal of Archaeological Science: Reports, 18, 1–10. https://doi.org/10.1016/j.jasrep.2017.12.007

McManus, E., Montgomery, J., Evans, J., Lamb, A., Brettell, R., & Jelsma, J. (2013). “To the Land or to the Sea”: Diet and Mobility in Early Medieval Frisia. In The Journal of Island and Coastal Archaeology (Vol. 8, Issue 2, pp. 255–277). Informa UK Limited. https://doi.org/10.1080/15564894.2013.787565

Milella, M., Gerling, C., Doppler, T., Kuhn, T., Cooper, M., Mariotti, V., Belcastro, M. G., Ponce de León, M. S., & Zollikofer, C. P. E. (2019). Different in death: Different in life? Diet and mobility correlates of irregular burials in a Roman necropolis from Bologna (Northern Italy, 1st–4th century CE). Journal of Archaeological Science: Reports, 27. https://doi.org/10.1016/j.jasrep.2019.101926

Minniti, C., Valenzuela-Lamas, S., Evans, J., & Albarella, U. (2014). Widening the market. Strontium isotope analysis on cattle teeth from Owslebury (Hampshire, UK) highlights changes in livestock supply between the Iron Age and the Roman period. Journal of Archaeological Science, 42(1), 305–314. https://doi.org/10.1016/j.jas.2013.10.008

Mion, L., Herrscher, E., Blondiaux, J., Binet, E., & Andre, G. (2016). Comportements alimentaires en Gaule du Nord : étude isotopique du site de l’Îlot de la Boucherie (iii e–v e siècles apr. J.-C.) à Amiens. Bulletins et Memoires de La Societe d’Anthropologie de Paris, 28(3–4), 155–175. https://doi.org/10.1007/s13219-016-0164-7

Moghaddam, N., Müller, F., & Lösch, S. (2018). A bioarchaeological approach to the Iron Age in Switzerland: stable isotope analyses (δ13C, δ15N, δ34S) of human remains. Archaeological and Anthropological Sciences, 10(5), 1067–1085. https://doi.org/10.1007/s12520-016-0441-x

Moghaddam, N., Müller, F., Hafner, A., & Lösch, S. (2016). Social stratigraphy in Late Iron Age Switzerland: stable carbon, nitrogen and sulphur isotope analysis of human remains from Münsingen. Archaeological and Anthropological Sciences, 8(1), 149–160. https://doi.org/10.1007/s12520-014-0221-4

Moles, A. C., Reade, H., Jourdan, A.-L., & Stevens, R. E. (2022). Stable isotopes reveal dietary shifts associated with social change in Hellenistic, Roman and Late Antique Knossos. In Journal of Archaeological Science: Reports (Vol. 45, p. 103609). Elsevier BV. https://doi.org/10.1016/j.jasrep.2022.103609

Montgomery, J., & Knüsel, C. J. (2011). Identifying the Origins of Decapitated Male Skeletons from 3 Driffield Terrace, York, Through Isotope AnalysisReflections of the Cosmopolitan Nature of Roman York in the Time of Caracalla. In The Bioarchaeology of the Human HeadDecapitation, Decoration, and Deformation (pp. 141–178). University Press of Florida. https://doi.org/10.5744/florida/9780813035567.003.0006

Montgomery, J., Evans, J. A., Powlesland, D., & Roberts, C. A. (2005). Continuity or colonization in Anglo-Saxon England? Isotope evidence for mobility, subsistence practice, and status at West Heslerton. American Journal of Physical Anthropology, 126(2), 123–138. https://doi.org/10.1002/ajpa.20111

Moore, J., Rose, A., Anderson, S., Evans, J., Nowell, G., Gröcke, D. R., Pashley, V., Kirby, M., & Montgomery, J. (2020). A multi-isotope (C, N, O, Sr, Pb) study of Iron Age and Roman period skeletons from east Edinburgh, Scotland exploring the relationship between decapitation burials and geographical origins. Journal of Archaeological Science: Reports, 29. https://doi.org/10.1016/j.jasrep.2019.102075

Müldner, G. (2013). Stable isotopes and diet: Their contribution to Romano-British research. Antiquity, 87(335), 137-149. https://doi.org/10.1017/S0003598X00048675

Müldner, G., & Richards, M. P. (2007). Stable isotope evidence for 1500 years of human diet at the city of York, UK. American Journal of Physical Anthropology, 133(1), 682–697. https://doi.org/10.1002/ajpa.20561

Müldner, G., Britton, K., & Ervynck, A. (2014). Inferring animal husbandry strategies in coastal zones through stable isotope analysis: New evidence from the Flemish coastal plain (Belgium, 1st-15th century AD). Journal of Archaeological Science, 41, 322–332. https://doi.org/10.1016/j.jas.2013.08.010

Müldner, G., Chenery, C., & Eckardt, H. (2011). The “Headless Romans”: Multi-isotope investigations of an unusual burial ground from Roman Britain. Journal of Archaeological Science, 38(2), 280–290. https://doi.org/10.1016/j.jas.2010.09.003

Murray, M. L., & Schoeninger, M. J. (1988). Diet, Status, and Complex Social Structure in Iron Age Central Europe: Some Contributions of Bone Chemistry. In Tribe and Polity in Late Prehistoric Europe (pp. 155–176). Springer US. https://doi.org/10.1007/978-1-4899-0777-6_7

Nafplioti, A. (2008). “Mycenaean” political domination of Knossos following the Late Minoan IB destructions on Crete: negative evidence from strontium isotope ratio analysis (87Sr/86Sr). Journal of Archaeological Science, 35(8), 2307–2317. https://doi.org/10.1016/j.jas.2008.03.006

Nafplioti, A. (2012). Late Minoan IB destructions and cultural upheaval on Crete: A bioarchaeological perspective. Population Dynamics in Prehistory and Early History: New Approaches Using Stable Isotopes and Genetics, edited by Elke Kaiser, Joachim Burger and Wolfram Schier, Berlin, Boston: De Gruyter, pp. 241-264. https://doi.org/10.1515/9783110266306.241

Nafplioti, A. (2016). Eating in prosperity: First stable isotope evidence of diet from Palatial Knossos. Journal of Archaeological Science: Reports, 6, 42–52. https://doi.org/10.1016/j.jasrep.2016.01.017

Nafplioti, A., Driessen, J., Schmitt, A., & Crevecoeur, I. (2021). Mobile (after-)lifeways: People at pre- and protopalatial Sissi (Crete). Journal of Archaeological Science: Reports, 35. https://doi.org/10.1016/j.jasrep.2020.102718

Nehlich, O. Fuller, B.T., Márquez-Grant, N., Richards, M.P. (2012). Investigation of Diachronic Dietary Patterns on the Islands of Ibiza and Formentera, Spain: Evidence from Sulfur Stable Isotope Ratio Analysis. American Journal of Physical Anthropology 149: 115-124.

Nehlich, O., Fuller, B. T., Jay, M., Mora, A., Nicholson, R. A., Smith, C. I., & Richards, M. P. (2011). Application of sulphur isotope ratios to examine weaning patterns and freshwater fish consumption in Roman Oxfordshire, UK. Geochimica et Cosmochimica Acta, 75(17), 4963–4977. https://doi.org/10.1016/j.gca.2011.06.009

Nicholls, R. A., Buckberry, J., Črešnar, M., Armit, I., Mason, P., & Koon, H. (2020). Interdisciplinarna študija človeških ostankov s starejšeželeznodobnega grobišča v Zagorju ob Savi. In Arheološki vestnik (Vol. 71). The Research Center of the Slovenian Academy of Sciences and Arts / Znanstvenoraziskovalni center Slovenske akademije znanosti in umetnosti (ZRC SAZU). https://doi.org/10.3986/av.71.17

Nicholls, R., Buckberry, J., Beaumont, J., Črešnar, M., Mason, P., Armit, I., & Koon, H. (2020). A carbon and nitrogen isotopic investigation of a case of probable infantile scurvy (6th–4th centuries BC, Slovenia). In Journal of Archaeological Science: Reports (Vol. 30, p. 102206). Elsevier BV. https://doi.org/10.1016/j.jasrep.2020.102206

Nieto-Espinet, A., Valenzuela-Lamas, S., Bosch, D., & Gardeisen, A. (2020). Livestock production, politics and trade: A glimpse from Iron Age and Roman Languedoc. Journal of Archaeological Science, Reports, 30(102077), 102077. doi:10.1016/j.jasrep.2019.102077

Nitsch, E., Andreou, S., Creuzieux, A., Gardeisen, A., Halstead, P., Isaakidou, V., Karathanou, A., Kotsachristou, D., Nikolaidou, D., Papanthimou, A., Petridou, C., Triantaphyllou, S., Valamoti, S. M., Vasileiadou, A., & Bogaard, A. (2017). A bottom-up view of food surplus: using stable carbon and nitrogen isotope analysis to investigate agricultural strategies and diet at Bronze Age Archontiko and Thessaloniki Toumba, northern Greece. World Archaeology, 49(1), 105–137. https://doi.org/10.1080/00438243.2016.1271745

O’Connell, T. C., Ballantyne, R. M., Hamilton-Dyer, S., Margaritis, E., Oxford, S., Pantano, W., Millett, M., & Keay, S. J. (2019). Living and dying at the Portus Romae. Antiquity, 93(369), 719–734. https://doi.org/10.15184/aqy.2019.64

Oelze, V. M., Koch, J. K., Kupke, K., Nehlich, O., Zäuner, S., Wahl, J., Weise, S. M., Rieckhoff, S., & Richards, M. P. (2012). Multi-isotopic analysis reveals individual mobility and diet at the early iron age monumental tumulus of magdalenenberg, germany. In American Journal of Physical Anthropology (Vol. 148, Issue 3, pp. 406–421). Wiley. https://doi.org/10.1002/ajpa.22063

Ortega, L. A., Guede, I., Zuluaga, M. C., Alonso-Olazabal, A., Murelaga, X., Niso, J., Loza, M., & Quirós Castillo, J. A. (2013). Strontium isotopes of human remains from the San Martín de Dulantzi graveyard (Alegría-Dulantzi, Álava) and population mobility in the Early Middle Ages. In Quaternary International (Vol. 303, pp. 54–63). Elsevier BV. https://doi.org/10.1016/j.quaint.2013.02.008

Paladin, A., Moghaddam, N., Stawinoga, A.E., Siebke, I., Depellegrin, V., Tecchiati, U., Lösch, S., Zink, A. (2020). Early medieval Italian Alps: reconstructing diet and mobility in the valleys. Archaeological and Anthropological Science 12. DOI: 10.1007/s12520-019-00982-6.

Panagiotopoulou, E., Montgomery, J., Nowell, G., Peterkin, J., Doulgeri-Intzesiloglou, A., Arachoviti, P., Katakouta, S., & Tsiouka, F. (2018). Detecting Mobility in Early Iron Age Thessaly by Strontium Isotope Analysis. European Journal of Archaeology, 21(4), 590–611. https://doi.org/10.1017/eaa.2017.88

Panagiotopoulou, E., van der Plicht, J., Papathanasiou, A., Voutsaki, S., Nikolaou, E., & Tsiouka, F. (2016). Isotopic (13C, 15N) investigation of diet and social structure in Early Iron Age Halos, Greece. Journal of Archaeological Science: Reports, 10, 212–220. https://doi.org/10.1016/j.jasrep.2016.09.020

Papathanasiou, A., Panagiotopoulou, E., Beltsios, K., Papakonstantinou, M. F., & Sipsi, M. (2013). Inferences from the human skeletal material of the Early Iron Age cemetery at Agios Dimitrios, Fthiotis, Central Greece. Journal of Archaeological Science, 40(7), 2924–2933. https://doi.org/10.1016/j.jas.2013.02.027

Pate, F. D., Henneberg, R. J., & Henneberg, M. (2016). Stable carbon and nitrogen isotope evidence for dietary variability at Ancient Pompeii, Italy. Mediterranean Archaeology and Archaeometry, 16(1), 127–133. https://doi.org/10.5281/zenodo.35526

Perry, M. A., Coleman, D., & Delhopital, N. (2008). Mobility and exile at 2nd century A.D. Khirbet edh-Dharih: Strontium isotope analysis of human migration in Western Jordan. Geoarchaeology, 23(4), 528–549. https://doi.org/10.1002/GEA.20230

Perry, M. A., Jennings, C., & Coleman, D. S. (2017). Strontium isotope evidence for long-distance immigration into the Byzantine port city of Aila, modern Aqaba, Jordan. Archaeological and Anthropological Sciences, 9(5), 943–964. https://doi.org/10.1007/S12520-016-0314-3

Petroutsa, E. I., & Manolis, S. K. (2010). Reconstructing Late Bronze Age diet in mainland Greece using stable isotope analysis. Journal of Archaeological Science, 37(3), 614–620. https://doi.org/10.1016/j.jas.2009.10.026

Pollard, A. M., Ditchfield, P., McCullagh, J. S. O., Allen, T. G., Gibson, M., Boston, C., Clough, S., Marquez-Grant, N., & Nicholson, R. A. (2011). “These boots were made for walking”: The isotopic analysis of a C 4 Roman inhumation from Gravesend, Kent, UK. American Journal of Physical Anthropology, 146(3), 446–456. https://doi.org/10.1002/ajpa.21602

Privat, K. L., O’connell, T. C., & Richards, M. P. (2002). Stable Isotope Analysis of Human and Faunal Remains from the Anglo-Saxon Cemetery at Berinsfield, Oxfordshire: Dietary and Social Implications. In Journal of Archaeological Science (Vol. 29, Issue 7, pp. 779–790). Elsevier BV. https://doi.org/10.1006/jasc.2001.0785

Prowse, T. L., Saunders, S. R., Schwarcz, H. P., Garnsey, P., Macchiarelli, R., & Bondioli, L. (2008). Isotopic and dental evidence for infant and young child feeding practices in an imperial roman skeletal sample. American Journal of Physical Anthropology, 137(3), 294–308. https://doi.org/10.1002/ajpa.20870

Prowse, T. L., Schwarcz, H. P., Garnsey, P., Knyf, M., Macchiarelli, R., & Bondioli, L. (2007). Isotopic evidence for age-related immigration to imperial Rome. American Journal of Physical Anthropology, 132(4), 510–519. https://doi.org/10.1002/ajpa.20541

Prowse, T., Schwarcz, H. P., Saunders, S., Macchiarelli, R., & Bondioli, L. (2004). Isotopic paleodiet studies of skeletons from Imperial Roman-age cemetery of Isola Sacra, Rome, Italy. Journal of Archaeological Science, 31(3), 259–272. https://doi.org/10.1016/j.jas.2003.08.008

Redfern, R. C., Gröcke, D. R., Millard, A. R., Ridgeway, V., Johnson, L., & Hefner, J. T. (2016). Going south of the river: A multidisciplinary analysis of ancestry, mobility and diet in a population from Roman Southwark, London. Journal of Archaeological Science, 74, 11–22. https://doi.org/10.1016/j.jas.2016.07.016

Redfern, R. C., Hamlin, C., & Athfield, N. B. (2010). Temporal changes in diet: A stable isotope analysis of late Iron Age and Roman Dorset, Britain. Journal of Archaeological Science, 37(6), 1149–1160. https://doi.org/10.1016/j.jas.2009.10.022

Redfern, R. C., Millard, A. R., & Hamlin, C. (2012). A regional investigation of subadult dietary patterns and health in late Iron Age and Roman Dorset, England. Journal of Archaeological Science, 39(5), 1249–1259. https://doi.org/10.1016/j.jas.2011.12.023

Redfern, R., Gowland, R., Millard, A., Powell, L., & Gröcke, D. (2018). ‘From the mouths of babes’: A subadult dietary stable isotope perspective on Roman London (Londinium). Journal of Archaeological Science: Reports, 19, 1030–1040. https://doi.org/10.1016/j.jasrep.2017.08.015

Reinberger, K. L., Reitsema, L. J., Kyle, B., Vassallo, S., Kamenov, G., & Krigbaum, J. (2021). Isotopic evidence for geographic heterogeneity in Ancient Greek military forces. PLoS ONE, 16(5 May). https://doi.org/10.1371/journal.pone.0248803

Reitsema, L. J., Kyle, B., & Vassallo, S. (2020). Food traditions and colonial interactions in the ancient Mediterranean: Stable isotope evidence from the Greek Sicilian colony Himera. Journal of Anthropological Archaeology, 57. https://doi.org/10.1016/j.jaa.2020.101144

Reitsema, L. J., Kyle, B., Koҫi, M., Horton, R. N., Reinberger, K. L., Lela, S., & Shehi, E. (2022). Bioarchaeological evidence for ancient human diet and migration at Epidamnus/Dyrrachion and Apollonia in Illyria, Albania. Archaeological and Anthropological Sciences, 14(5), 1–25. https://doi.org/10.1007/S12520-022-01553-Y

Ricci, P., Sirignano, C., Altieri, S., Pistillo, M., Santoriello, A., & Lubritto, C. (2016). Paestum dietary habits during the Imperial period: Archaeological records and stable isotope measurement. Acta IMEKO, 5(2), 26–32. https://doi.org/10.21014/acta_imeko.v5i2.334

Riccomi, G., Minozzi, S., Zech, J., Cantini, F., Giuffra, V., & Roberts, P. (2020). Stable isotopic reconstruction of dietary changes across Late Antiquity and the Middle Ages in Tuscany. In Journal of Archaeological Science: Reports (Vol. 33, p. 102546). Elsevier BV. https://doi.org/10.1016/j.jasrep.2020.102546

Richards, M. P., Fuller, B. T., & Hedges, R. E. M. (2001). Sulphur isotopic variation in ancient bone collagen from Europe: implications for human palaeodiet, residence mobility, and modern pollutant studies. Earth and Planetary Science Letters, 191(3–4), 185–190. https://doi.org/10.1016/S0012-821X(01)00427-7

Richards, M. P., Hedges, R. E. M., Molleson, T. I., & Vogel, J. C. (1998). Stable Isotope Analysis Reveals Variations in Human Diet at the Poundbury Camp Cemetery Site. In Journal of Archaeological Science (Vol. 25). https://doi.org/10.1006/jasc.1998.0307

Rissech, C., Pujol, A., Christie, N., Lloveras, L., Richards, M. P., & Fuller, B. T. (2016). Isotopic reconstruction of human diet at the Roman site (1st-4th c. AD) of Carrer Ample 1, Barcelona, Spain. Journal of Archaeological Science: Reports, 9, 366–374. https://doi.org/10.1016/j.jasrep.2016.08.020

Rutgers, L. v., van Strydonck, M., Boudin, M., & van der Linde, C. (2009). Stable isotope data from the early Christian catacombs of ancient Rome: new insights into the dietary habits of Rome’s early Christians. Journal of Archaeological Science, 36(5), 1127–1134. https://doi.org/10.1016/j.jas.2008.12.015

Salazar-García, D. C., Colominas, L., & Jordana, X. (2022). Food for the soul and food for the body. Studying dietary patterns and funerary meals in the Western Roman Empire: An anthropological and archaeozoological approach. In L. Bondioli (Ed.), PLOS ONE (Vol. 17, Issue 8, p. e0271296). Public Library of Science (PLoS). https://doi.org/10.1371/journal.pone.0271296

Salazar-García, D. C., Romero, A., García-Borja, P., Subirà, M. E., & Richards, M. P. (2016). A combined dietary approach using isotope and dental buccal-microwear analysis of human remains from the Neolithic, Roman and Medieval periods from the archaeological site of Tossal de les Basses (Alicante, Spain). Journal of Archaeological Science: Reports, 6, 610–619. https://doi.org/10.1016/j.jasrep.2016.03.002

Salesse, K., Dufour, É., Balter, V., Tykot, R. H., Maaranen, N., Rivollat, M., Kharobi, A., Deguilloux, M. F., Pemonge, M. H., Brůžek, J., & Castex, D. (2021). Far from home: A multi-analytical approach revealing the journey of an African-born individual to imperial Rome. Journal of Archaeological Science: Reports, 37. https://doi.org/10.1016/j.jasrep.2021.103011

Sandias, M., & Müldner, G. (2015). Diet and herding strategies in a changing environment: Stable isotope analysis of Bronze Age and Late Antique skeletal remains from Ya’amūn, Jordan. Journal of Archaeological Science, 63, 24–32. https://doi.org/10.1016/j.jas.2015.07.009

Saragoça, P., Maurer, A.F. Šoberl, L., da Conceição Lopes, M., Alfenim, R., Leandro, I., Umbelino, C., Fernandes, T., Valente, M.J., Ribeiro, S., Santos, J.F., Janeiro, A.I., Dias Barrocas, C. (2016). Stable isotope and multi-analytical investigation of Monte da Cegonha: A Late Antiquity population in southern Portugal. Journal of Archaeological Science: Reports 9: 728-742.

Scheeres, M., Knipper, C., Hauschild, M., Schönfelder, M., Siebel, W., Vitali, D., Pare, C., & Alt, K. W. (2013). Evidence for “Celtic migrations”? Strontium isotope analysis at the early La Tène (LT B) cemeteries of Nebringen (Germany) and Monte Bibele (Italy). Journal of Archaeological Science, 40(10), 3614–3625. https://doi.org/10.1016/j.jas.2013.05.003

Schmidt, J., Kwok, C., & Keenleyside, A. (2016). Infant feeding practices and childhood diet at Apollonia Pontica: Isotopic and dental evidence. American Journal of Physical Anthropology, 159(2), 284–299. https://doi.org/10.1002/ajpa.22874

Schuh, C., & Makarewicz, C. A. (2016). Tracing residential mobility during the Merovingian period: An isotopic analysis of human remains from the Upper Rhine Valley, Germany. In American Journal of Physical Anthropology (Vol. 161, Issue 1, pp. 155–169). Wiley. https://doi.org/10.1002/ajpa.23017

Schulting, R. J., le Roux, P., Gan, Y. M., Pouncett, J., Hamilton, J., Snoeck, C., Ditchfield, P., Henderson, R., Lange, P., Lee-Thorp, J., Gosden, C., & Lock, G. (2019). The ups & downs of Iron Age animal management on the Oxfordshire Ridgeway, south-central England: A multi-isotope approach. Journal of Archaeological Science, 101, 199–212. https://doi.org/10.1016/j.jas.2018.09.006

Schweissing, M. M., & Grupe, G. (2003). Stable strontium isotopes in human teeth and bone: A key to migration events of the late Roman period in Bavaria. Journal of Archaeological Science, 30(11), 1373–1383. https://doi.org/10.1016/S0305-4403(03)00025-6

Schweissing, M. M., & Grupe, G. (2003). Tracing migration events in man and cattle by stable strontium isotope analysis of appositionally grown mineralized tissue. International Journal of Osteoarchaeology, 13(1–2), 96–103. https://doi.org/10.1002/oa.652

Scirè Calabrisotto, C., Fedi, M. E., Taccetti, F., Benvenuti, M., Chiarantini, L., & Quaglia, L. (2009). Radiocarbon reveals the age of two precious tombs in the etruscan site of Populonia-Baratti (Tuscany). Radiocarbon, 5, 3: 915-922. https://doi.org/10.1017/S0033822200033981

Scorrano, G., Brilli, M., Martínez-Labarga, C., Giustini, F., Pacciani, E., Chilleri, F., Scaldaferri, F., Gasbarrini, A., Gasbarrini, G., & Rickards, O. (2014). Palaeodiet reconstruction in a woman with probable celiac disease: A stable isotope analysis of bone remains from the archaeological site of Cosa (Italy). American Journal of Physical Anthropology, 154(3), 349–356. https://doi.org/10.1002/ajpa.22517

Sengeløv, A., van de Wijdeven, G., Snoeck, C., Laffoon, J., de Hond, R., Gnade, M., & Waters-Rist, A. (2020). Understanding the post-Archaic population of Satricum, Italy: A bioarchaeological approach. Journal of Archaeological Science: Reports, 31. https://doi.org/10.1016/j.jasrep.2020.102285

Shaw, H., Montgomery, J., Redfern, R., Gowland, R., & Evans, J. (2016). Identifying migrants in Roman London using lead and strontium stable isotopes. Journal of Archaeological Science, 66, 57–68. https://doi.org/10.1016/j.jas.2015.12.001

Sheridan, S. G., & Gregoricka, L. A. (2015). Monks on the move: Evaluating pilgrimage to byzantine St. Stephen’s monastery using strontium isotopes. American Journal of Physical Anthropology, 158(4), 581–591. https://doi.org/10.1002/ajpa.22827

Siebke, I., Furtwängler, A., Steuri, N., Hafner, A., Ramstein, M., Krause, J., & Lösch, S. (2020). Crops vs. animals: regional differences in subsistence strategies of Swiss Neolithic farmers revealed by stable isotopes. In Archaeological and Anthropological Sciences (Vol. 12, Issue 10). Springer Science and Business Media LLC. https://doi.org/10.1007/s12520-020-01122-1

Sirignano, C., Sologestoa, I.G., Ricci, P., García-Collado, M.I., Altieri, S., Quirós Castillo, J.A., Lubritto, C. (2014). Animal husbandry during Early and High Middle Ages in the Basque Country (Spain). Quaternary International 346: 138-148.

Sisma-Ventura, G., Zohar, I., Sarkar, A., Bhattacharyya, K., Zidane, A., Gilboa, A., … Sivan, D. (2015). Oxygen isotope composition of Sparidae (sea bream) tooth enamel from well-dated archaeological sites as an environmental proxy in the East Mediterranean: A case study from Tel Dor, Israel. Journal of Archaeological Science, 64, 46–53. doi:10.1016/j.jas.2015.10.004

Sofeso, C., Vohberger, M., Wisnowsky, A., Päffgen, B. and Harbeck, M. (2012). Verifying archaeological hypotheses: Investigations on origin and genealogical lineages of a privileged society in Upper Bavaria from Imperial Roman times (Erding, Kletthamer Feld). In: Kaiser, E., Burger, J. and Schier, W. ed. Population Dynamics in Prehistory and Early History: New Approaches Using Stable Isotopes and Genetics. Berlin, Boston: De Gruyter, pp. 113-130. https://doi.org/10.1515/9783110266306.113

Sorrentino, R., Bortolini, E., Lugli, F., Mancuso, G., Buti, L., Oxilia, G., Vazzana, A., Figus, C., Serrangeli, M. C., Margherita, C., Penzo, A., Gruppioni, G., Gottarelli, A., Jochum, K. P., Belcastro, M. G., Cipriani, A., Feeney, R. N. M., & Benazzi, S. (2018). Unravelling biocultural population structure in 4th/3rd century BC Monterenzio Vecchio (Bologna, Italy) through a comparative analysis of strontium isotopes, non-metric dental evidence, and funerary practices. PLoS ONE, 13(3). https://doi.org/10.1371/journal.pone.0193796

Sparkes, H. A. (2017). Taking a Bite out of History: Hellenistic Dietary Reconstruction and Population Mobility at the Site of New Halos, Thessaly, Greece. [PhD thesis. University of Alberta]. https://doi.org/10.7939/R34M91P3J

Stark, R. J., Emery, M. v., Schwarcz, H., Castex, D., & Prowse, T. L. (2022). Assessing Gallo-Roman mobility at the Rue Jacques Brel necropolis site (1st to 3rd c. CE), France. Journal of Archaeological Science: Reports, 43. https://doi.org/10.1016/j.jasrep.2022.103470

Stark, R. J., Emery, M. v., Schwarcz, H., Sperduti, A., Bondioli, L., Craig, O. E., & Prowse, T. (2020). Imperial Roman mobility and migration at Velia (1st to 2nd c. CE) in southern Italy. Journal of Archaeological Science: Reports, 30. https://doi.org/10.1016/j.jasrep.2020.102217

Stevens, R. E., Lightfoot, E., Allen, T., & Hedges, R. E. M. (2012). Palaeodiet at Eton College Rowing Course, Buckinghamshire: Isotopic changes in human diet in the Neolithic, Bronze Age, Iron Age and Roman periods throughout the British Isles. Archaeological and Anthropological Sciences, 4(3), 167–184. https://doi.org/10.1007/s12520-012-0089-0

Stevens, R. E., Lightfoot, E., Hamilton, J., Cunliffe, B. W., & Hedges, R. E. M. (2013). One for the master and one for the dame: Stable isotope investigations of Iron Age animal husbandry in the Danebury Environs. Archaeological and Anthropological Sciences, 5(2), 95–109. https://doi.org/10.1007/s12520-012-0114-3

Stevens, R. E., Lightfoot, E., Hamilton, J., Cunliffe, B., & Hedges, R. E. M. (2010). Stable isotope investigations of the Danebury hillfort pit burials. In Oxford Journal of Archaeology (Vol. 29, Issue 4, pp. 407–428). Wiley. https://doi.org/10.1111/j.1468-0092.2010.00355.x

Sykes, N. J., Baker, K. H., Carden, R. F., Higham, T. F. G., Hoelzel, A. R., & Stevens, R. E. (2011). New evidence for the establishment and management of the European fallow deer (Dama dama dama) in Roman Britain. Journal of Archaeological Science, 38(1), 156–165. https://doi.org/10.1016/j.jas.2010.08.024

Sykes, N. J., White, J., Hayes, T. E., & Palmer, M. R. (2006). Tracking animals using strontium isotopes in teeth: the role of fallow deer (Dama dama) in Roman Britain. Antiquity, 80, 310: 948-959. https://doi.org/10.1017/S0003598X00094539

Tafuri, M. A., Goude, G., & Manzi, G. (2018). Isotopic evidence of diet variation at the transition between classical and post-classical times in Central Italy. Journal of Archaeological Science: Reports, 21, 496–503. https://doi.org/10.1016/j.jasrep.2018.08.034

Tanasi, D., Tykot, R. H., Vianello, A., & Hassam, S. (2017). Stable isotope analysis of the dietary habits of a Greek community in Archaic Syracuse (Sicily): a pilot study. Science and Technology of Archaeological Research, 3(2), 466–477. https://doi.org/10.1080/20548923.2018.1441695

Toffolo MB, Fantalkin A, Lemos IS, Felsch RCS, Niemeier W-D, et al. (2013) Towards an Absolute Chronology for the Aegean Iron Age: New Radiocarbon Dates from Lefkandi, Kalapodi and Corinth. PLoS ONE 8(12): e83117. https://doi.org/10.1371/journal.pone.0083117

Toyne, J. M., Čelhar, M., & Nystrom, K. C. (2021). Liburnian lunches: New stable isotope data for the Iron Age community of Nadin‐Gradina, Croatia. In International Journal of Osteoarchaeology (Vol. 32, Issue 1, pp. 241–257). Wiley. https://doi.org/10.1002/oa.3059

Trentacoste, A., Lightfoot, E., le Roux, P., Buckley, M., Kansa, S. W., Esposito, C., & Gleba, M. (2020). Heading for the hills? A multi-isotope study of sheep management in first-millennium BC Italy. Journal of Archaeological Science: Reports, 29. https://doi.org/10.1016/j.jasrep.2019.102036

Triantaphyllou, S., Nikita, E., & Kador, T. (2015). Exploring mobility patterns and biological affinities in the southern Aegean: first insights from early bronze age eastern Crete. Annual of the British School at Athens, 110, 3–25. https://doi.org/10.1017/S0068245415000064

Triantaphyllou, S., Richards, M. P., Touchais, G., Philippa-Touchais, A., & Voutsaki, S. (2006). Analyses of Middle Helladic Skeletal Material from Aspis, Argos, 2. Stable Isotope Analysis of Human Remains. Bulletin de Correspondance Hellénique, 130(2), 627–637. https://doi.org/10.3406/bch.2006.7430

Triantaphyllou, S., Richards, M. P., Zerner, C., & Voutsaki, S. (2008). Isotopic dietary reconstruction of humans from Middle Bronze Age Lerna, Argolid, Greece. Journal of Archaeological Science, 35(11), 3028–3034. https://doi.org/10.1016/j.jas.2008.06.018

Trias, M. C., Rosselló, J. G., Molina, D. J., Santacreu, D. A., & Van Strydonck, M. (2014). Matching Data: Analyzing the Chronological Use Sequence in the Iron Age Necropolis of the Staggered Turriform of Son Ferrer (Balearic Islands, Spain). In Radiocarbon (Vol. 56, Issue 2, pp. 361–374). Cambridge University Press (CUP). https://doi.org/10.2458/56.17498

Triozzi, B. (2021). A Biocultural Study of the Populations of the Lower Pescara Valley and Its Hinterland: Health, Diet, and Identity in the 6th-4th c. BC in Abruzzo (Italy). [PhD Thesis. The University of Sheffield]. uk.bl.ethos.831207

Valenzuela-Lamas, S., Jiménez-Manchón, S., Evans, J., López, D., Jornet, R., & Albarella, U. (2016). Analysis of seasonal mobility of sheep in Iron Age Catalonia (north-eastern Spain) based on strontium and oxygen isotope analysis from tooth enamel: First results. Journal of Archaeological Science, Reports, 6, 828–836. doi:10.1016/j.jasrep.2015.08.042

Valenzuela-Lamas, Silvia, Orengo, H. A., Bosch, D., Pellegrini, M., Halstead, P., Nieto-Espinet, A., … Jornet-Niella, R. (2018). Shipping amphorae and shipping sheep? Livestock mobility in the north-east Iberian peninsula during the Iron Age based on strontium isotopic analyses of sheep and goat tooth enamel. PloS One, 13(10), e0205283. doi:10.1371/journal.pone.0205283

van Strydonck, M., Ervynck, A., Vandenbruaene, M., & Boudin, M. (2009). Anthropology and14C Analysis of Skeletal Remains from Relic Shrines: An Unexpected Source of Information for Medieval Archaeology. In Radiocarbon (Vol. 51, Issue 2, pp. 569–577). Cambridge University Press (CUP). https://doi.org/10.1017/s0033822200055934

Varalli, A., Moggi-Cecchi, J., & Goude, G. (2022). A multi-proxy bioarchaeological approach reveals new trends in Bronze Age diet in Italy. Scientific Reports, 12(1), 12203. doi:10.1038/s41598-022-15581-0

Varano, S., de Angelis, F., Battistini, A., Brancazi, L., Pantano, W., Ricci, P., Romboni, M., Catalano, P., Gazzaniga, V., Lubritto, C., Santangeli Valenzani, R., Martínez-Labarga, C., & Rickards, O. (2020). The edge of the Empire: diet characterization of medieval Rome through stable isotope analysis. Archaeological and Anthropological Sciences, 12(8). https://doi.org/10.1007/s12520-020-01158-3

Veselka, B., Capuzzo, G., Annaert, R., Mattielli, N., Boudin, M., Dalle, S., Hlad, M., Sabaux, C., Salesse, K., Sengeløv, A., Stamataki, E., Tys, D., Vercauteren, M., Warmenbol, E., De Mulder, G., & Snoeck, C. (2021). Divergence, diet, and disease: the identification of group identity, landscape use, health, and mobility in the fifth- to sixth-century AD burial community of Echt, the Netherlands. In Archaeological and Anthropological Sciences (Vol. 13, Issue 6). Springer Science and Business Media LLC. https://doi.org/10.1007/s12520-021-01348-7

Vika, E. (2009). Strangers in the grave? Investigating local provenance in a Greek Bronze Age mass burial using δ34S analysis. Journal of Archaeological Science, 36(9), 2024–2028. https://doi.org/10.1016/j.jas.2009.05.022

Vika, E. (2011). Diachronic dietary reconstructions in ancient Thebes, Greece: Results from stable isotope analyses. Journal of Archaeological Science, 38(5), 1157–1163. https://doi.org/10.1016/j.jas.2010.12.019

Vika, E., & Theodoropoulou, T. (2012). Re-investigating fish consumption in Greek antiquity: Results from δ13C and δ15N analysis from fish bone collagen. Journal of Archaeological Science, 39(5), 1618–1627. https://doi.org/10.1016/j.jas.2012.01.016

Wallace, M. P., Jones, G., Charles, M., Fraser, R., Heaton, T. H. E., & Bogaard, A. (2015). Stable Carbon Isotope Evidence for Neolithic and Bronze Age Crop Water Management in the Eastern Mediterranean and Southwest Asia. https://doi.org/10.1371/journal.pone.0127085

Wang, X., Zhang, X., Fan, A., Sampson, A., Wu, X., Gao, J., Huang, F., & Jin, Z. (2019). Strontium isotopic evidence for the provenance of occupants and subsistence of Sarakenos Cave in prehistoric Greece. Quaternary International, 508, 13–22. https://doi.org/10.1016/j.quaint.2018.10.009

Winter-Schuh, C., & Makarewicz, C. A. (2018). Isotopic evidence for changing human mobility patterns after the disintegration of the Western Roman Empire at the Upper Rhine. In Archaeological and Anthropological Sciences (Vol. 11, Issue 6, pp. 2937–2955). Springer Science and Business Media LLC. https://doi.org/10.1007/s12520-018-0702-y

Wong, M., Brandt, J. R., Ahrens, S., Jaouen, K., Bjørnstad, G., Nauman, E., Wenn, C. C., Kiesewetter, H., Laforest, C., Hagelberg, E., Lam, V. C., & Richards, M. (2018). Pursuing pilgrims: Isotopic investigations of Roman and Byzantine mobility at Hierapolis, Turkey. Journal of Archaeological Science: Reports, 17, 520–528. https://doi.org/10.1016/j.jasrep.2017.12.005

Zavodny, E., Culleton, B. J., McClure, S. B., Kennett, D. J., & Balen, J. (2017). Minimizing risk on the margins: Insights on Iron Age agriculture from stable isotope analyses in central Croatia. Journal of Anthropological Archaeology, 48, 250–261. doi:10.1016/j.jaa.2017.08.004

Zavodny, E., McClure, S. B., Welker, M. H., Culleton, B. J., Balen, J., & Kennett, D. J. (2019). Scaling up: Stable isotope evidence for the intensification of animal husbandry in Bronze-Iron Age Lika, Croatia. Journal of Archaeological Science, Reports, 23, 1055–1065. doi:10.1016/j.jasrep.2018.10.008
