# Supplementary material for: Introducing Isotòpia: A stable isotope database for Classical Antiquity
Source: PLoS One. 2024 Jun 3;19(6):e0293717. doi: 10.1371/journal.pone.0293717 (PMC11146721; doi:10.1371/journal.pone.0293717)
Supplement: S3 Appendix — (DOCX) [file pone.0293717.s004.docx]

S4. Spatiotemporal groupings using KernelTimeR

As data points, we considered each chronological range per archaeological site. Using site coordinates and associated temporal ranges, an average 3-dimensional spatiotemporal Kernel density is estimated from samples across dating uncertainty[1]. The clustering method implemented in the R package Mclust [2] is applied to site coordinates to define clusters irrespective of their dating. Within each cluster and for each point the average kernel density across time was calculated. The point within a cluster with the highest average kernel density was chosen as a centroid since higher densities are expected for points with better dating precision and temporal coverage. Finally, cluster membership was re-assigned so that each site was placed within the temporal group having the closest centroid.

References

1. Wand MP, Jones MC. Multivariate plug-in bandwidth selection. Comput Stat. 1994;9: 97–116.

2. Scrucca L, Fop M, Murphy TB, Raftery AE. mclust 5: Clustering, Classification and Density Estimation Using Gaussian Finite Mixture Models. R J. 2016;8: 289–317.
